# Cancer survivor preferences for breast cancer follow-up care: a discrete choice experiment

**DOI:** 10.1007/s11764-024-01629-9

**Published:** 2024-06-14

**Authors:** Sameera Senanayake, Sanjeewa Kularatna, Fiona Crawford-Williams, David Brain, Michelle Allen, Ruvini M. Hettiarachchi, Nicolas H. Hart, Bogda Koczwara, Carolyn Ee, Raymond J. Chan

**Affiliations:** 1 Australian Centre for Health Services Innovation (AusHSI), Centre for Healthcare Transformation, School of Public Health & Social Work, Queensland University of Technology (QUT), 60 Musk Avenue, Victoria Park Road, Kelvin Grove, Brisbane, QLD 4059 Australia; 2 Duke-NUS Medical School, Health Services and Systems Research, Singapore, Singapore; 3 Caring Futures Institute, College of Nursing and Health Sciences, Flinders University, Adelaide, SA Australia; 4 Centre for the Business and Economics of Health (CBEH), University of Queensland, Brisbane, QLD Australia; 5 Faculty of Health, Human Performance Research Centre, INSIGHT Research Institute, University of Technology Sydney (UTS), Sydney, NSW Australia; 6 Cancer and Palliative Care Outcomes Centre, School of Nursing, Faculty of Health, Queensland University of Technology (QUT), Brisbane, QLD Australia; 7 Exercise Medicine Research Institute, School of Medical and Health Science, Edith Cowan University, Perth, WA Australia; 8 Institute for Health Research, University of Notre Dame Australia, Perth, WA Australia; 9 College of Medicine and Public Health, Flinders Health and Medical Research Institute, Flinders University, Adelaide, SA Australia; 10 Flinders Centre for Innovation in Cancer, Flinders Medical Centre, Adelaide, SA Australia; 11 NICM Health Research Institute, Western Sydney University, Sydney, NSW Australia

**Keywords:** Breast cancer, Survivorship, Discrete choice experiment, Patient-preference

## Abstract

**Purpose:**

To identify the key attributes of breast cancer follow-up care models preferred by cancer survivors in Australia.

**Methods:**

A discrete choice experiment (DCE) was conducted to elicit preferences for attributes of breast cancer follow-up care. Respondents were presented with two hypothetical scenarios, known as choice sets, and asked to select a preference. Respondents were individuals living in Australia who were diagnosed with breast cancer within the past five years prior to survey completion and were recruited through the Breast Cancer Network of Australia and other community or consumer networks. Latent class modelling (LCM) approach under a random utility framework was used for the analysis.

**Results:**

123 breast cancer survivors completed the DCE survey. LCA revealed two latent classes, those with older age and lower quality of life (class 1) and younger women with higher quality of life (class 2). Class 2 preferred a care team comprising specialists, nurses and GPs and emphasised the importance of shared survivorship care plans. Class 1 remained neutral regarding the team’s composition but was notably concerned about the out-of-pocket costs per consultation, a finding not seen in Class 2.

**Conclusions:**

Age and quality of life status are associated with patient preference for types and attributes of breast cancer follow-up care. The health system can work towards enhancing flexibility of follow-up care delivery, ultimately achieving person-centred care.

Implications for cancer survivors.

Efforts need to be made by policymakers to ensure consumer preferences are taken into consideration to implement tailored person-centred follow-up care pathways.

**Supplementary information:**

The online version contains supplementary material available at 10.1007/s11764-024-01629-9.

## Introduction

Breast cancer remains the most frequently diagnosed malignancy in Australian women, with an annual incidence exceeding 20,000 cases in 2021 [1, 2]. Fortunately, survival rates of women with early breast cancer are high, with a 5-year relative survival of 91.5% [2]. However, a considerable proportion of breast cancer survivors experience significant physical and psychological consequences from cancer treatment as well as substantial out-of-pocket healthcare expenditures within the first five years post-diagnosis [3, 4]. Long-term follow-up care is advised for breast cancer survivors to ameliorate the impact of physical and psychological consequences, and is typically provided in the hospital setting by cancer specialists. Despite guidelines and models of follow-up care recommending the involvement of general practitioners (GPs) to optimise quality of care and ensure care suits the needs of breast cancer survivors following primary treatment [5], these models are not routinely implemented at present. Incorporating shared-care models between specialists and GPs into the delivery of follow-up care could potentially mitigate healthcare system costs, enhance patient experiences and are as safe and effective as specialist-led models [6, 7].

Understanding preferences of breast cancer survivors for follow-up care models is essential to facilitate planning, organisation, financing and delivering care in a more efficient, suitable and sustainable way [8]. It is crucial that cancer survivors are given an opportunity to articulate their preferences and that these preferences are communicated to decision-makers to inform care model redesign [9]. Amongst methodologies for assessing preferences of people, discrete choice experiments (DCE) are increasingly recognised as an effective tool for comprehending decision-making processes between hypothetical alternative scenarios in healthcare [10, 11]. Specifically, respondents are presented with a succession of hypothetical situations that differ in their characteristics, such as wait times, proximity to care, cost and care provider, and are required to select their preferred choice from each scenario. DCE methodologies are particularly advantageous when investigators seek to understand the relative significance of various attributes and their impact on decision-making [12]. By analysing the choices made by participants, decision-makers can determine the preferences and trade-offs individuals are prepared to make when presented with different options [10]. This method of measuring preferences is essential to explore what works for cancer survivors and advocate for improvements to cancer care within a complex health system. Accordingly, this study aimed to understand key attributes of breast cancer follow-up care models preferred by breast cancer survivors through DCE.

## Method

A discrete choice experiment is a research technique where participants are presented with hypothetical scenarios and asked to choose their preferred option from a set of alternatives. Each scenario consists of various attributes with different levels, providing a detailed picture of what factors are most important to respondents. In some DCEs, the options are labelled with specific names (like brand names), whilst in non-labelled ones, only the attributes are shown without specific identifiers. Participants typically go through multiple repeated choice task, comparing and selecting their preferred option several times. This method helps researchers understand preferences and trade-offs people are willing to make.

An online DCE survey was administered to a sample of female breast cancer survivors who had completed treatment within the last five years in Australia. Participants were predominantly recruited through the Breast Cancer Network Australia Review and Survey Group (a national group of 1374 Australian women living with breast cancer who are interested in receiving invitations to participate in research and members of one of the largest consumer advocacy networks for people with breast cancer in Australia). Additionally, the survey was advertised through existing networks of the research team. Emails were sent out to potential participants in August 2022, with reminders sent in November 2022 and January 2023 and the survey closed in 2023. Ethical approval was obtained for this study from the Queensland University of Technology Human Research Ethics Committee on 28/10/2021 (ID: 4567- HE31).

The DCE was a non-labelled survey with respondents presented with two hypothetical scenarios at a time, known as choice sets. The selection of the final set of attributes and levels for this DCE was based on a review of the literature, focus groups with consumers and health service providers, a quantitative structured prioritisation exercise, and an expert panel discussion [6, 13]. The breast cancer follow-up care models were described by five attributes and their corresponding levels (Table 1).

**Table 1:** Included attributes and their associated levels

Attribute description	Levels
Care team providing cancer follow-up care	• Medical specialists and breast cancer nurse • Medical specialists, breast cancer nurse and General Practitioner
Allied health (e.g. exercise and dietetics) and supportive care	• 5 allied health and 10 psychology • 10 allied health plus 10 psychology • 15 allied health plus 10 psychology
Survivorship care plan (detailed document outlining all care arrangements)	• No survivorship care plan • Survivorship care plan is developed and shared with the healthcare team • Survivorship care plan is developed and shared with the healthcare team and the patient
Travel to follow-up appointment/s	• No travel (telehealth) • Travel up to 50 km for every follow-up appointment • Travel for more than 50 km for every follow appointment
Out-of-pocket costs to the patient per appointment	• $0 • $100 • $200

In the study, the concept of “follow-up care” was conceptualised as a comprehensive care model for individuals who have completed cancer treatment. This model aims to enhance the overall well-being of cancer survivors by including various aspects, such as surveillance for cancer recurrence and screening for secondary cancers, monitoring of physical late effects resulting from cancer and/or treatment, management of psychosocial concerns, promotion of health and addressing comorbidities. The second attribute, “allied health” professionals, were defined according to the Medicare scheme. This includes Aboriginal health workers or Aboriginal and Torres Strait Islander health practitioners, audiologists, chiropractors, diabetes educators, dietitians, exercise physiologists, mental health workers, occupational therapists, osteopaths, physiotherapists, podiatrists, psychologists and speech pathologists. The number indicated in the levels corresponds to the number of sessions or consultations provided by these professionals. In the scope of this study, the term “Survivorship Care Plan” denotes a comprehensive document shared amongst the survivor, oncologist and the broader care team. This document encompasses various elements, namely: a summary of the cancer treatment received, a clearly outlined schedule for follow-up appointments and screening tests, including the contact information of the healthcare professionals involved in the treatment and ongoing care, a compilation of potential symptoms to be vigilant of and the potential long-term side effects to anticipate, identification of medical, emotional, psychological or social needs post-treatment, along with strategies for their management, clarification of the roles and responsibilities of different members within the healthcare team and the appropriate points of contact in case of concerns, and recommendations for adopting a healthy lifestyle post-treatment.

**Fig. 1:**
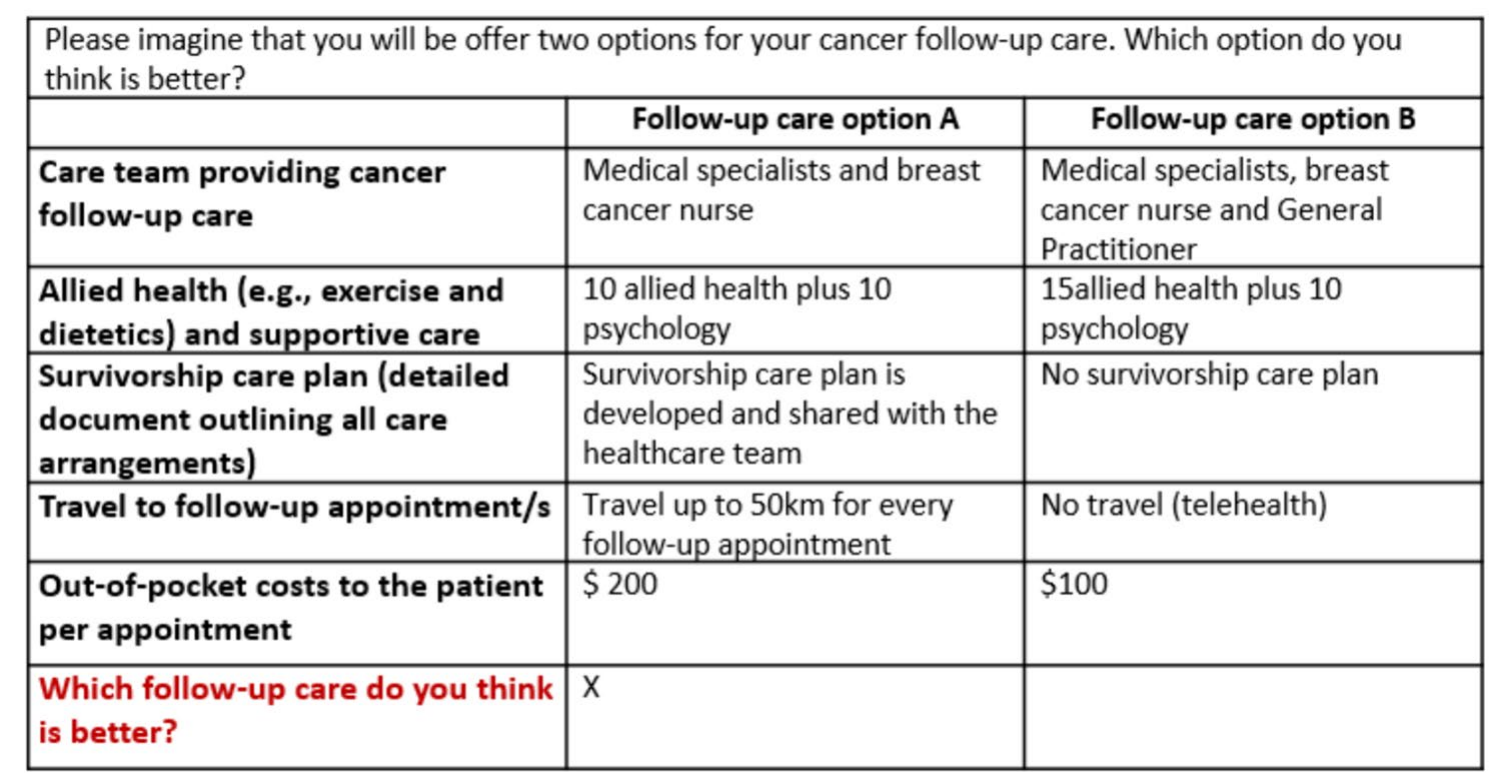
Example of a choice task seen by respondents

The online DCE survey consisted of three sections. First, the respondents received information on completing a DCE task and were shown a sample task (Fig. 1). Demographic information, such as gender, age and education level were also collected to summarise the characteristics of participants. The second section consisted of DCE tasks including one repeated and one dominant choice task to assess response validity. The third-choice task, which was one of the ten choice tasks, was repeated at the end of the ten main choice tasks. The proportion of participants who provided identical responses to both tasks was used as an estimate for assessing the internal reliability and consistency of responses. Additionally, a choice task featuring an apparent dominant alternative was administered following the main DCE tasks. The proportion of participants who correctly identified this dominant option served as a secondary estimate for evaluating the internal reliability and consistency of responses[14, 15]. In the third section, respondents completed the 5-level EuroQol 5-dimension (EQ 5D-5L) multi-attribute utility instrument. EQ 5D-5L is a generic quality-of-life instrument that has been used amongst people with cancer [16], and recently published Australian-specific tariffs were used to estimate the EQ 5D-5L utility scores [17].

The final attribute list and corresponding levels would result in 26,244 (3^8^ × 2^2^) possible choice tasks. Since it is not feasible to present all possible combinations to all respondents, 20 choice tasks were selected using a fractional factorial design, which makes up the choice sets. The main aim of using a fractional factorial design was to have a manageable number of choice tasks whilst maximising the design’s statistical efficiency [18]. Prior research [12, 19] indicates that individuals are capable of effectively responding to ten-choice sets simultaneously. Therefore, the fractional factorial design in this study was divided into two blocks, with each participant being presented with only ten of the possible 20 choice tasks.

The final DCE design used a Bayesian D-efficient design with normally distributed priors generated using Ngene software [20]. The priors were derived through pilot testing with 28 respondents. Since there was no existing data available regarding the coefficients for the various attributes, non-informative priors—small positive or negative priors, or zero priors—were utilised to develop the D-efficient design for the pilot (Supplementary Table 1 and Supplementary Fig. 1). The design was constructed based on a priori hypotheses. The final Bayesian D-efficient design was optimised using the Modified Federov algorithm employing 1000 Halton draws (Supplementary Table 1 and Supplementary Fig. 2). The Bayesian D-error for the design is 0.1355. In addition to the D-error, the final design was assessed based on attribute level overlap—where attribute levels are present in both choice tasks, and attribute level balance—the distribution of attribute levels across the two choice tasks. A lower degree of attribute level overlap and equal distribution of attribute levels are indicative of an optimal DCE design (Supplementary Table 2).

To accommodate individual preferences, a latent class modelling (LCM) approach under a random utility framework was used for the analysis [21]. The random utility framework assumes that the participants chose the alternative that maximised their utility. LCA identifies unobservable, or “latent,” subgroups within participants’ preferences. LCA postulates that within each latent class, preferences are homogeneous, yet these preferences differ distinctively across classes [22]. The assignment of respondents to these classes is probabilistic, estimating the likelihood of each respondent belonging to a specific class based on their responses. Incorporating respondent characteristics enhances the precision of these probabilistic class assignments. The selection of an optimal number of latent classes was informed by statistical fit measures, such as the Akaike Information Criterion (AIC) and log-likelihood values, ensuring the identification of genuine preference patterns, rather than mere stochastic variations (Supplementary Table 3). Socio-demographics and other data collected as part of the online survey were analysed using descriptive statistics. Mixed logit and multinomial logit regression models were also fitted, but they were inferior to the latent class model (Supplementary Table 3).

Furthermore, we estimated the willingness to pay (WTP) values for each attribute level. The WTP metric, commonly utilised in DCE, quantifies the monetary value respondents assign to specific attributes or their changes. The WTP is estimated by dividing the utility coefficient of a given attribute by the utility coefficient of the cost attribute. This ratio, termed the marginal rate of substitution, indicates the monetary value respondents associate with a unit change in the attribute in question. These WTP estimates indicates the relative importance respondents assign to distinct healthcare attributes and reflect the monetary trade-offs they are predisposed to make in their healthcare decisions.

All analyses were conducted using the NLOGIT 5 software [23].

## Results

There were 123 participants who completed the online DCE survey (approximately 8% response rate). The demographic characteristics of the sample are presented in Table 2. Most respondents (57%) were between 46 to 65 years old and resided in Queensland (59%). The mean EQ 5D-5L utility score was 0.91 (SD 0.11). The median time taken to respond to the survey was 15 min (IQR 11 to 20 min), which was within the expected time from the pilot. The dominant and repeat tasks were correct in 115 (91%) and 114 (90%) of the responses, respectively.

**Table 2:** Sample characteristics

Variable	Categories	*N* = 126; number (%)
**Age in years**	26–45	17 (13.5%)
	46–55	32 (25.4%)
	56–65	40 (31.7%)
	66–75	32 (25.4%)
	75 +	5 (4.0%)
**State**	Queensland	74 (58.7%)
	New South Wales	26 (20.6%)
	Victoria	12 (9.5%)
	Western Australia	5 (4.0%)
	South Australia	4 (3.2%)
	Australian Capital Territory (ACT)	4 (3.2%)
	Northern Territory	1 (0.8%)
**EQ 5D 5L utility**	Mean	0.91
	SD	0.11

In the LCA, we discerned two distinct latent classes within the respondent dataset. LCA enables the probabilistic categorisation of respondents into groups based on observed variables, which, in this case, are age and reported quality of life. Our findings indicated that respondents of an older age bracket, coupled with a reported lower quality of life, had a higher probability of association with latent class 1. Conversely, respondents who were relatively younger and reported a higher quality of life demonstrated a higher likelihood of alignment with latent class 2. It is pivotal to note that LCA does not provide deterministic assignment to a class. Instead, it offers a probabilistic measure of a respondent’s affiliation to one class over another, based on their attributes. In terms of sample distribution, latent class 1 encompassed 33% of the respondents, whereas latent class 2 constituted a larger segment, capturing 64% of the sample.

The model results are presented in Table 3. Differences in preferences between the two classes were evident regarding the care team providing cancer follow-up care, the availability of a survivorship plan, and the out-of-pocket costs per appointment for breast cancer survivors. There was no difference in preferences between classes concerning numbers of allied health and psychology appointments or travel to follow-up appointments. Class 2 respondents preferred a care team consisting of medical specialists, breast cancer nurses and GPs for providing cancer follow-up care, whilst Class 1 respondents showed indifference to the care team providers. Class 2 respondents preferred the development and sharing of a survivorship care plan with the healthcare team and cancer survivor, followed by the plan being shared only with the healthcare team, as opposed to not having a survivorship plan at all. Meanwhile, Class 1 respondents showed indifference towards the development of the survivorship plan. Class 1 respondents were highly sensitive to the out-of-pocket costs of an appointment, whilst this concern was not shared by those in Class 2.

**Table 3:** Model results

	**Class 1** **(coefficient and 95% CI)**	**Class 2** **(coefficient and 95% CI)**
Care team providing cancer follow-up care		
* Medical specialists and breast cancer nurse*	Reference	Reference
* Medical specialists, breast cancer nurse and General Practitioner*	− 0.73 [− 1.63, 0.17]	0.69 [0.27, 1.11]
Allied health (e.g. exercise and dietetics) and supportive care		
* 5 allied health and 10 psychology*	Reference	Reference
* 10 allied health plus 10 psychology*	− 0.52 [− 1.44, 0.4]	0.06 [− 0.32, 0.44]
* 15 allied health plus 10 psychology*	− 0.19 [− 1.12, 0.73]	− 0.21 [− 0.61, 0.19]
Survivorship care plan (detailed document outlining all care arrangements)		
* No survivorship care plan*	Reference	Reference
* Survivorship care plan is developed and shared with the healthcare team*	− 1.1 [− 2.26, 0.06]	1.62 [1.21, 2.04]
* Survivorship care plan is developed and shared with the healthcare team and the patient*	− 0.54 [− 1.75, 0.67]	2.25 [1.73, 2.78]
Travel to follow-up appointment/s		
* No travel (telehealth)*	1.05 [0.01, 2.09]	0.59 [0.18, 1.01]
* Travel up to 50 km for every follow-up appointment*	− 0.52 [− 1.55, 0.51]	0.54 [0.16, 0.92]
* Travel for more than 50 km for every follow appointment*	Reference	Reference
Out-of-pocket costs to the patient per appointment (per $100)	− 3.07 [− 3.85, − 2.3]	− 0.01 [− 0.23, 0.21]
Age	0.015 [− 0.028, 0.058]	− 0.002 [− 0.015, 0.011]
EQ 5D 5L utility values	0.56 [− 1.62, 2.74]	1.24 [− 0.33, 2.82]
Class probability (95% CI)	33.1% [24.5%, 42.8%]	66.4% [57.2%, 75.5%]

The willingness to pay (WTP) estimates are presented in Table 4. Respondents’ preference for a care team consisting of medical specialists, breast cancer nurses and general practitioners was highlighted by their willingness to pay AUD $57 more to receive treatment from this care team compared to one consisting of only medical specialists and breast cancer nurses. The respondents were willing to pay AUD $132 for a survivorship care plan developed by the healthcare team and were willing to pay higher (AUD $221) for a survivorship plan when they were also involved in its development. They were willing to pay AUD $68 to avoid travelling, thus utilising telehealth services.

**Table 4:** Average willingness to pay (WTP) for different attributes

	**Willingness to pay in AUD [95% CI]**
Care team providing cancer follow-up care	
* Medical specialists and breast cancer nurse*	
* Medical specialists, breast cancer nurse and General Practitioner*	$-57 [$-99, $-16]
Allied health (e.g. exercise and dietetics) and supportive care	
* 5 allied health and 10 psychology*	Reference
* 10 allied health plus 10 psychology*	$4 [$-41, $50]
* 15 allied health plus 10 psychology*	$23 [$-25, $71]
Survivorship care plan (detailed document outlining all care arrangements)	
* No survivorship care plan*	Reference
* Survivorship care plan is developed and shared with the healthcare team*	$-132 [$-182, $-82]
* Survivorship care plan is developed and shared with the healthcare team and the patient*	$-221 [$-277, $-165]
Travel to follow-up appointment/s	
* No travel (telehealth)*	$-68 [$-112, $-24]
* Travel up to 50 km for every follow-up appointment*	$-42 [$-88, $3]
* Travel for more than 50 km for every follow appointment*	Reference

## Discussion

This study is the first to investigate patient preferences for breast cancer follow-up with a focus on shared-care models focussing on incorporation of survivorship care plans and primary care providers including general practitioners and allied health professionals. The key findings suggest that there are differences in preferences between older survivors with lower quality of life and younger survivors with higher quality of life in regards to members of the care team and development of a survivorship care plan. The findings are in line with existing evidence which suggests that many cancer survivors highly value involvement in survivorship care planning and appreciate a multi-disciplinary team to support them including acute and primary care providers [3, 24–26]. This study is significant as the need for patient-centred, holistic survivorship care and long-term follow-up is recognised [5, 8]. These findings need to be considered by health services when tailoring follow-up care to individuals.

The current findings demonstrate that certain patient characteristics are associated with quite different priorities, needs and preferences when it comes to follow-up care. Younger women care more about being involved in the development of personalised care and a comprehensive care team, including general practitioners, perhaps because this has become the social norm [3], whereas older women are more impacted by out-of-pocket costs, potentially given their restricted income. Regardless of demographic cohort, the number of allied health and supportive care appointments was not a relevant factor for breast cancer survivors when choosing a follow-up care model, and all preferred less travel to appointments. This is important information for those designing and delivering health services and for policy makers. This finding should be interpreted as survivors not valuing allied health care. The options available for selection compared two levels of intensity in terms of number of sessions and did not compare allied health sessions versus no allied health involvement at all.

Previous literature suggests that shared-care models of follow-up support holistic care and have equivalent patient outcomes and satisfaction to specialist-led follow-up models [6]. Thus, Cancer Australia recommends shared-care follow-up for people with early breast cancer [5]. However, there remain health system barriers to implementing shared-care models, and these guidelines are not being routinely implemented in health services. If cancer care is to become truly patient-centred, then where resources are available, we recommend that health services invest in follow-up models that patients value. The WTP values give an indication of how much survivors value the various attributes of survivorship care. Lessons learnt may be taken from other disciplines. For example, a variety of models including specialist-led, shared-care and midwifery-led care that have been successfully implemented in antenatal care for many years [27]. Further advances in system-level implementation, policy and advocacy will be required to afford cancer survivors the same level of flexibility, achieving truly patient-centred care [6].

This study focused predominantly on women diagnosed with early breast cancer, with a relatively small sample that was mainly based in Queensland. However, we did not collect data on ethnic and cultural background, postcode or rural/regional remote status. Thus, such data may be helpful for understanding if survivors from a wider range of geographic locations and cultural or socioeconomic backgrounds have different preferences. Given participants were predominantly recruited through the Breast Cancer Network Australia Review and Survey Group, there is a risk that women with higher educational and health literacy are over-represented. It is uncertain as to how this potential bias might lead to certain direction for the results. Future research should examine the relationships between a range of social determinants of health and patient preference in shared- survivorship care. Furthermore, understanding of consumer preferences amongst those diagnosed with metastatic disease is needed as a population group who are likely to remain on treatment for the remainder of their lives, and may have differing needs in relation to survivorship care planning and primary care involvement. Another potential limitation of this study is the high quality of life reported by participants, which may introduce bias. This could result in overly positive perceptions of follow-up care, underestimating barriers faced by individuals with lower quality of life. Consequently, the findings may not fully represent the experiences of all cancer survivors. Future studies should aim for a more diverse sample.

This study should be replicated with survivors with the varying factors abovementioned, as well as different tumour types who may present different clinical need. Future research should also attempt to explore how policymakers and health administrators may use patient preferences and WTP values for informing policy decision making achieving a patient-centre health care system [9].

## Conclusion

Age and quality of life status are associated with patient preference for types and attributes of breast cancer follow-up care. Working towards a person-centred care, policy makers and clinician researchers should work in collaboration to enhance flexibility of follow-up care delivery enabling cancer survivors to access care that is required, with attributes that are valued by them.

## Supplementary Information

Below is the link to the electronic supplementary material.

Supplementary file1 (DOCX 229 KB)

## Data Availability

The datasets generated during and/or analysed during the current study are available from the corresponding author on reasonable request.
