# Supplementary material for: Cancer survivor preferences for breast cancer follow-up care: a discrete choice experiment
Source: J Cancer Surviv. 2024 Jun 14;20(1):315–22. doi: 10.1007/s11764-024-01629-9 (PMC12906538; doi:10.1007/s11764-024-01629-9)
Supplement: Supplementary file 1 — Supplementary file1 (DOCX 229 KB) [file 11764_2024_1629_MOESM1_ESM.docx]

**Supplementary material**

***Designing DCE choice tasks – pilot survey (Step 2)***

A multinomial logit model based on D-efficient fractional factorial design criteria (using the D-error value) was used to develop 20 pair-wise choice tasks using the design software Ngene (Supplementary figure 1).

Evidence indicates that respondents can efficiently handle ten choice sets at a time ^(8, 13)^. Therefore, the fractional factorial design was divided into two blocks so that a respondent would only answer ten from the 20 choice tasks in the fractional factorial design. Blocking is an accepted statistical technique in a DCE design that ensures an equal number of respondents per block^(14)^. We used the modified Federov algorithm to develop the D-efficient design, which is known to develop designs with attribute level balance and no dominant choice tasks^(15)^.

In the absence of prior information on the coefficients of the different attributes, small positive or negative priors or zero priors (non-informative priors) were used to design the D-efficient design based on the following a priori hypotheses. Breast cancer survivors prefer:

- care from a ‘medical specialist, breast cancer nurse, and General Practitioner’ led team.
- more allied health and supportive care appointments
- development of a survivorship plan, which is shared among all stakeholders
- less travelling
- less out-of-pocket cost


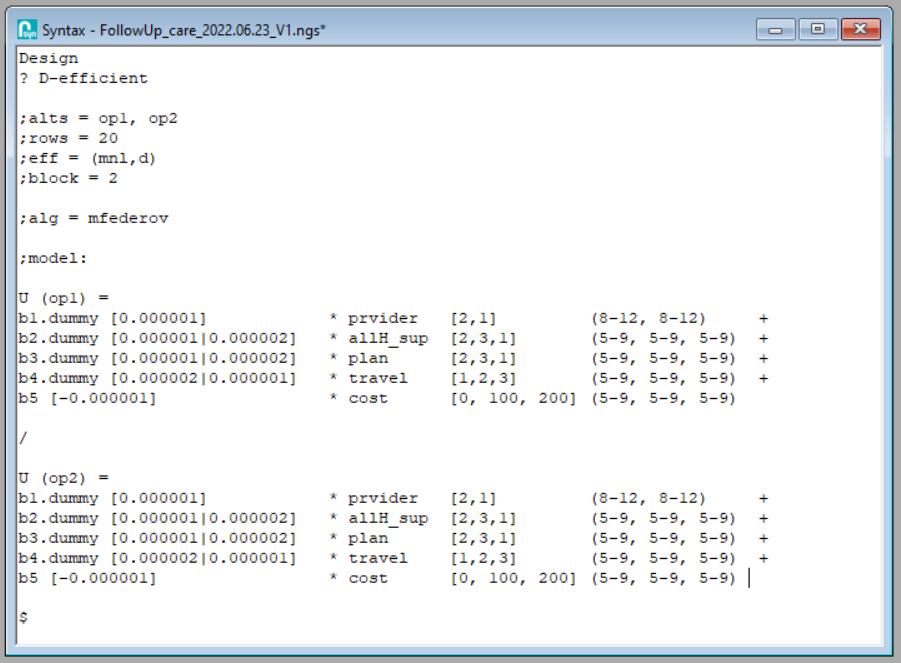


**Supplementary figure 1:** NGene code used to design the DCE choice tasks foe the pilot survey

**Supplementary table 1:** Priors used to design the choice tasks for the pilot and the final survey

| **Attribute** | **Priors used in the Ngene design** | |
| --- | --- | --- |
|  | **DCE design used for the pilot survey** | **DCE design used for the final survey** |
| Care team providing cancer follow-up care |  |  |
| - *Medical specialists and breast cancer nurse* | Reference | Reference |
| - *Medical specialists, breast cancer nurse and General Practitioner* | 0.000001 | 1.72996 |
| Allied health (e.g., exercise and dietetics) and supportive care |  |  |
| - *5 allied health and 10 psychology* | Reference | Reference |
| - *10 allied health plus 10 psychology* | 0.000001 | 0.41755 (SD: 0.37215) |
| - *15 allied health plus 10 psychology* | 0.000002 | 0.36294 (SD:0.40395) |
| Survivorship care plan (detailed document outlining all care arrangements) |  |  |
| - *No survivorship care plan* | Reference | Reference |
| - *Survivorship care plan is developed and shared with the healthcare team* | 0.000001 | 0.82490 |
| - *Survivorship care plan is developed and shared with the healthcare team and the patient* | 0.000002 | 2.55663 |
| Travel to follow-up appointment/s |  |  |
| - *No travel (telehealth)* | 0.000002 | 0.38502 (SD: 0.41850) |
| - *Travel up to 50km for every follow-up appointment* | 0.000001 | 0.44567 (SD: 0.38046) |
| - *Travel for more than 50 km for every follow appointment* | Reference | Reference |
| Out-of-pocket costs to the patient per appointment |  |  |
| - *$0* | 0.000001  (Continuous scale) | -0.00949 |
| - *$100* |  |  |
| - *$200* |  |  |
|  |  |  |
| *Measures of efficiency* |  |  |
| D-error | 0.0849 | 0.1355 |

**
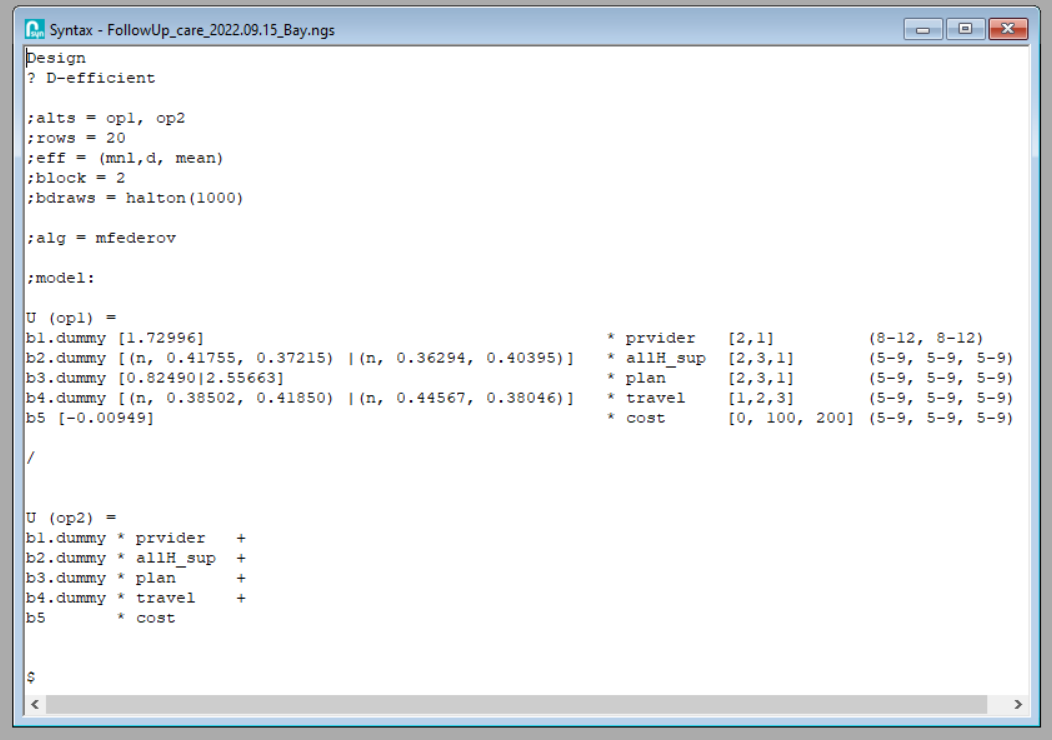
**

**Supplementary figure 1:** NGene code used to design the DCE choice tasks foe the pilot survey

**Supplementary Table 2:** Description of the final D efficient design

|  | **DCE design used for the final survey** | |
| --- | --- | --- |
| Number of rows | 20 | |
| Blocks | 2 | |
|  |  | |
| *Attribute overlap* |  | |
| Care team providing cancer follow-up care | 3/20 | |
| Allied health (e.g., exercise and dietetics) and supportive care | 0/20 | |
| Survivorship care plan (detailed document outlining all care arrangements) | 0/20 | |
| Travel to follow-up appointment/s | 0/20 | |
| Out-of-pocket costs to the patient per appointment | 2/20 | |
| ***Attribute level balance*** | **DCE design used for the pilot study** | |
|  | ***Choice task 1*** | ***Choice task 2*** |
| Care team providing cancer follow-up care |  |  |
| *Medical specialists and breast cancer nurse* | 9/20 | 8/20 |
| *Medical specialists, breast cancer nurse and General Practitioner* | 11/20 | 12/20 |
| Allied health (e.g., exercise and dietetics) and supportive care |  |  |
| *5 allied health and 10 psychology* | 7/20 | 8/20 |
| *10 allied health plus 10 psychology* | 5/20 | 5/20 |
| *15 allied health plus 10 psychology* | 8/20 | 7/20 |
| Survivorship care plan (detailed document outlining all care arrangements) |  |  |
| *No survivorship care plan* | 8/20 | 7/20 |
| *Survivorship care plan is developed and shared with the healthcare team* | 6/20 | 8/20 |
| *Survivorship care plan is developed and shared with the healthcare team and the patient* | 6/20 | 5/20 |
| Travel to follow-up appointment/s |  |  |
| *No travel (telehealth)* | 6/20 | 6/20 |
| *Travel up to 50km for every follow-up appointment* | 8/20 | 5/20 |
| *Travel for more than 50 km for every follow appointment* | 6/20 | 9/20 |
| Out-of-pocket costs to the patient per appointment |  |  |
| *$0* | 8/20 | 8/20 |
| *$100* | 5/20 | 5/20 |
| *$200* | 7/20 | 7/20 |

**Supplementary table 3:** Model fit criteria for the three models fitted for the data

| **Model** | **Log Likelihood** | **AIC/N** |
| --- | --- | --- |
| Multinomial logit | -810.8 | 1.301 |
| Panel mixed Multinomial logit | -781.9 | 1.266 |
| Latent Class Panel Model (2 classes) | -709.3 | 1.172 |
| Latent Class Panel Model (3 classes) | -712.5 | 1.179 |
| Latent Class Panel Model (4 classes) | The LCM does not converge as the estimated variance matrix of estimates is singular | |
